# Institutional Needs

**Published:** 1915-07-24

**Authors:** 


### Institutional Needs.

THE "BUFFALO" MEAT AND VEGETABLE CHOPPER.

The Avonmore Engineering Co., Ltd., Kingsway House, Kingsway, W.C., have placed on the market an ingenious meat, food, and vegetable chopper, known as the "Buffalo," designed to secure fine chopping without mashing the food. To cut raw meat fine, without waste of the juices or the leaving of lumps; to mix mayonnaise; to work at high speed; to mince without waste ingredients for croquettes, or cheese or nut dishes: these are what are secured by the machine's revolving bowl, which brings the meat, or other substance to be cut, under the comb. This comb may be detached, so as to make the bowl easy to clean after use. If we take No. 16 outfit as an example its technical points are described thus:—Bowl of cutter, 17 in. in diameter; two knives; capacity, 12 lb. of raw meat in five minutes, cooked meat and vegetables in two minutes; large enough to feed 500 people; 1-h.p. motor; net weight, 435 lb.; weight (crated), 485 lb.; weight (boxed), 585 lb.; box measurements, 47 in. by 28 in. by 49 in. The price-list is for alternating current, 2 or 3 phase, 60 cycle, 110, 220 or 440 volts, $160; floor space, 36 in. wide by 24 in. deep. For alternating current, single phase, 60 cycle, 110, 220 or 440 volts, $200; floor space, 44 in. wide by 24 in. deep. For direct current, 110, 220 or 500 volts, $165; floor space, 43 in. wide by 24 in. deep. Machines of any size and capacity are obtainable. Kitchen specialities in notable variety are also manufactured by the Avonmore Engineering Company. For instance, the firm's "Vortex" dish-washing machine (two- or three-tank type), comprising three baskets for china, one for cups, and one for silver. Another ingenious mechanical labour-saving device is the Company's "Acme" cutlery renovator, in which the chief aim of the design is, by the automatic adjustment of the rolls, to minimise the wear and tear of the blades during cleaning. As each knife varies in thickness and size, it adjusts the rolls to fit it, and, under capable handling, is quick, gentle, and easy to operate, and does not occupy much table space. A feature of the "Acme" is that the knife is visible throughout the process. Ingenuity and care are displayed in the Avonmore designs and material, and institutional managers could hardly do better than inspect their catalogues before placing their orders.

A NEW BANDAGE-WINDER.

In our issue for July 3 there appeared a descriptive notice of a new bandage-winder invented by Mr. T. J. Carr, of Newcastle. Our criticism of certain details of this machine appears to the inventor not to have done adequate justice to one or two of the novel features he claims for it. We understand that there is no need for tapering of the spindle or for making this square; in fact, the machine was designed to get away from the square spindle and, as the inventor states, "consequent telescoping." We cannot admit, however, that in actual practice square spindles are any great disadvantage. Mr. Carr now writes to say that on the face of the disc there is a projection which engages the end of the bandage and prevents any slip. This projection does not apparently figure on the detailed plan supplied to us as the sole source of our information. This is an omission of some importance for which the draughtsman of the plan must, presumably, be blamed. This particular bandage-winder will, no doubt, perform its work efficiently, but its superiority over other designs can only be estimated by practical experience, an opportunity for which may one day be available.

